# How social organizations participate in social governance in China: Official media’s attention distribution analysis (1949–2021)

**DOI:** 10.1371/journal.pone.0295322

**Published:** 2024-01-11

**Authors:** Huangjuan Liu, Fujun Ma, Xiaoman Chen

**Affiliations:** 1 School of Public Administration, Southwestern University of Finance and Economics, Wenjiang District, Chengdu City, Sichuan Province, China; 2 School of Physical Education, XiHua University, PiDu District, Chengdu City, Sichuan Province, China; Sichuan Agricultural University, CHINA

## Abstract

The attitude of the Chinese government towards social organizations (SOs) is crucial, as it affects the management rule and development tendency of SOs. To research the rule of SOs’ participation in social governance in China, this study used a new historical perspective, the institutional development perspective, to conduct its exploration. This perspective provides an accurate measure of the reality of the SOs’ participation, as it involves a mixed research methodology using continuous data from 73 years of reports and content mining, as well as topic clustering analysis to reveal a macroscopic and multi-line picture. Using a co-word analysis of hundreds of reports, from 1949–2021, in the *People’s Daily*, an official newspaper of the Communist Party of China, this study quantified changes in intensity, emotion, and content regarding social organization participation in social governance through topic distribution. Three trends were revealed: (1) “social-oriented character” and “organized-oriented character” were identified during the change in SOs; (2) the extent of being managed gradually strengthened and shifted from the Communist Youth League of China to the Community Party of China; (3) the goals of SOs shifted from general to innovated function in special charitable organizations. The institutional development perspective can complement the focus event perspective, including a new method, co-word analysis, to examine official Chinese media and validate the Administrative Absorption of Society (AAS) theory by identifying two lines of topic clustering trends. The attention distribution analysis in official media from an institutional development perspective can help explore the role of official media reports in analyzing the allocation of national attention and provide new analytical methods for big data mining to establish the social and organizational natures of SOs to optimize their roles. It offers a basis for modern social governance policy innovation in China.

## Introduction

The United Nations characterizes non-governmental organizations as “social organizations” (SO); however, the term’s scope can be broadened to include other organizations [1]. In China, SOs are organizations not belonging to the following types: the government, political party, businesses, and family. There are three main types of SOs in China: private non-profit groups, foundations, and social service groups [2]. Social organizations have existed in the country for more than 70 years, during which historical changes regarding their development and management have been reflected in official media reports.

To study the impact of media agendas on public opinions, the agenda setting theory (AST) has been used in text analyses of media reports [3]; in particular, the focus event perspective—which entails assessing the developmental stages of SOs by important events that attract widespread attention and discussion in society—has been used in attention analyses to explain significant changes in management policy for SOs [4]. However, the focus event perspective cannot explain the attention distribution of official media. In China, official media reports are the primary sources of information and are perceived as more trustworthy than popular media [5]; therefore, these reports cannot be explained by popular media theories. Official media reflects its own attention distribution—as evidenced by analyses of the intensity, emotion, and content of reports—and of the policymakers, as conveyed by the selection and dissemination of topics guiding public opinion and discussion. Research has demonstrated that posts from official media and public comments differed by themes [6]. The media’s framing can affect public opinion and policy debates, with different types of newspapers presenting different frameworks [7]. Therefore, we can assume that the attention distribution of official media conveys the government and/or national perspectives, and is characterized by state control, planning, and development [8].

Unlike AST and the focus event perspective, a constructive perspective can explain the complex relationship between the content and the attention distribution of official media, including the national attention distribution. Additionally, a constructive perspective based on the national governance and policy formulation can use content analysis to measure and reveal the natural development of the official media’s attention distribution. Specifically, this institutional development perspective can be applied to explain the laws of SOs participating in social governance.

The existing AST cannot explain the impact of the state’s active intervention in official media and largely negates the broader role of SOs in social governance. Limiting analyses to the focus event perspective and point-to-point connections ignores the nation-building view and may not reflect reality. Thus, researchers must modify the measurement tools, re-examine the process and effects of SOs’ participation in social governance, and supplement AST from a national perspective. In addition to public, media, and policy attention, official media facilitates comprehensive attention distribution. Text mining can reveal this function and help examine how it is anchored in the institutional development. The attitude of the Chinese government towards SOs is crucial, because it affects the existence and development of SOs in the standardization of management, through their registration, annual inspection, and evaluation standards. Under the “big government” style of government-society relationship, this study provides a comprehensive historical picture. The Chinese government’s position toward SOs can be explored by analyzing excerpts from *People’s Daily* as an elementary reflection of the will of the state. Notably, China has a distinctive system for its SOs, which is combined with civil society organizations and official background social organization. Accordingly, an analysis of co-words reported by official media was conducted to examine how SOs participate in social governance in China.

## Literature review

The management and development of SOs in the Chinese system differs from the democratic practice of the West and from general theories of development, such as multi-center governance theory, in which public services are provided by multiple subjects [9]. To date, studies have identified changes in SOs participating in social governance in China using focus event analyses.

### Focus event perspective

A “focus event” is a social event viewed as a critical motivator for policy field change. Notably, these events can reduce or increase the speed of policy change [10]. The cross-sectional perspective of focus event analysis examines turning points. There are two main types of focus event analyses: a case analysis of SO participation in major social events, and a milestone analysis of new stages of SO development [11]. For example, SO management changed in response to China’s reform and opening-up [12–14] and can be categorized into management styles that were prevalent throughout of the periods before and after the reform and opening-up. This kind of research provides a holistic view of the development of SOs. However, the practical focus event perspective is limited by the text, which is heavily dependent on the quality of major events—hence, a quantitative analysis cannot be conducted. Accordingly, a long-term retrospective observation is needed.

### Institutional construction perspective

Institutional development analysis assumes that an institution comprises rules, norms, and procedures [15]. The institutional perspective focuses on how management ideas and tools change. Many policymakers and researchers believe that ideology, value choice, economic system reform, and political system reform are crucial factors impacting change in SO management [16]. From the institutional development perspective of top-down management, the state’s attention can be used to gauge the effect of SO management, which can serve as a policy tool for control intensity, development promotion, and technology development; this is often reflected in policy documents and official media reports [10, 17, 18]. The institutional development perspective can reveal the SO management logic from the institutional level [19]. For example, regarding the relationship between the government and SOs, some scholars distinguished between “classified control,” “regulatory control,” and “empowerment control” [18].

These aspects of the institutional development perspective make it ideal for SO analysis for two main reasons. First, media attention closely tracks institutional changes [20, 21], and Chinese media agendas and political agendas show a high degree of congruence [22]. The distribution of attention varies according to societal issues and is positively correlated with institutional changes. Second, media attention changes remain highly consistent with changes in local public issue practices. These trends are well-known; as early as 1958, Norton Long introduced the concept of “local issue setting” in newspapers, and in 1972, Maxwell McCombs and Donald Shaw’s empirical study showed that locally discussed issues were significantly correlated with issues highlighted by local news media. The distribution of popular attention was gradually aligned with that of policymakers through media [3].

Ghanem divided the media framework into four dimensions: news topics (content choice), external performance (word length and newspaper layout), cognitive attributes (details), and emotional attributes (tone of the article) [23]. The attention content, attention intensity, overall attention rhythm, and the macro-affective tendency picture of public issues can describe a “world image” and “simulative environment” shaped by the media.

By ordering time clues and high-frequency topic words, this study focuses on the attention distribution of SOs to reduce information fragmentation, revealing SO background, organizational structure, social function, role positioning, and ecosystem. With a special focus on policymakers’ attention intensities, emotions, and attention targets related to SO in different stages, this study offers an overview of Chinese SO management and development.

**Fig 1:**
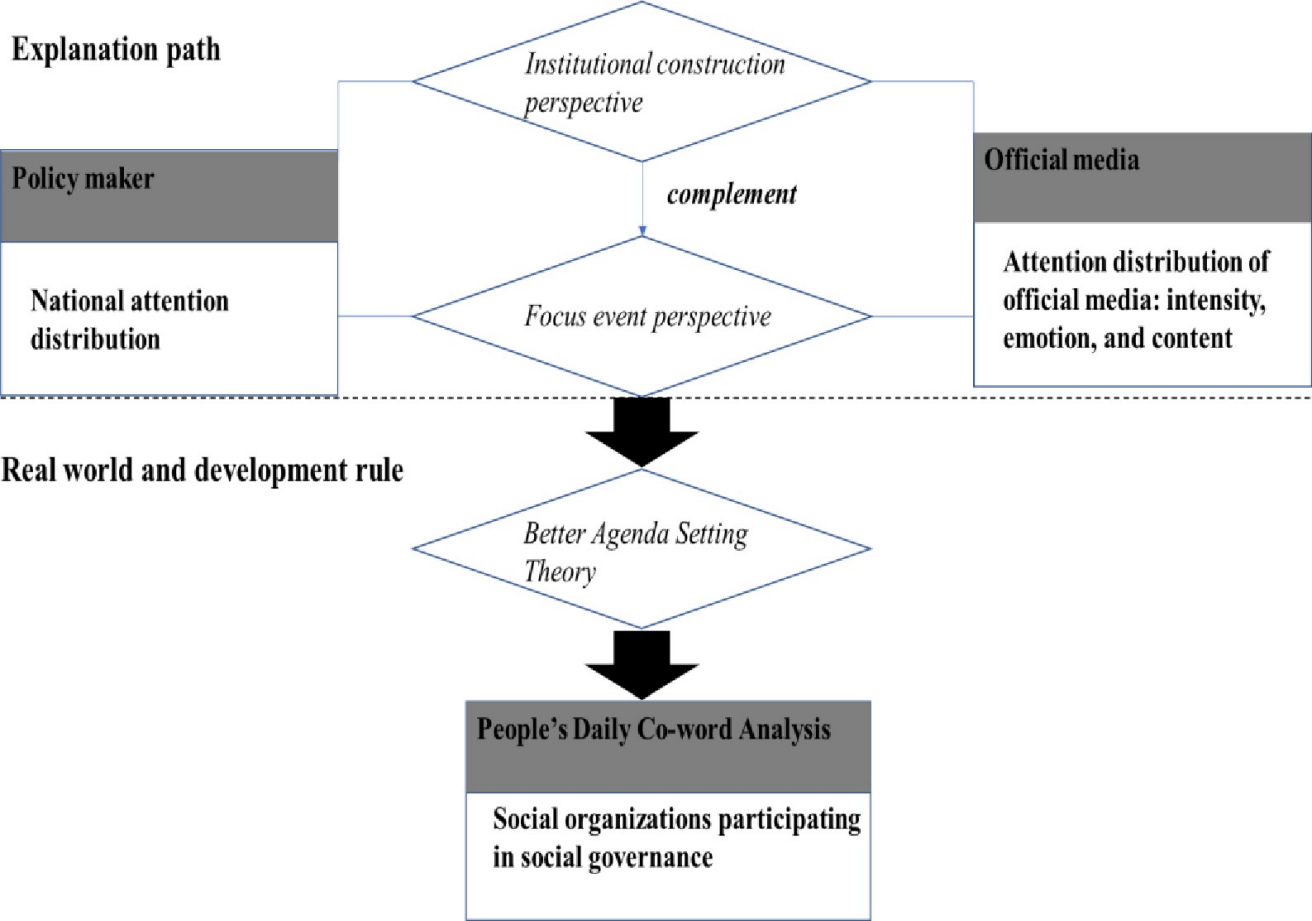
Research framework.

Policy formulation, implementation, and change require guidance from the central level, increased attention from functional departments, participation of experts and scholars, and public feedback. When these distinct roles are combined, they yield comprehensive power. In China, this kind of comprehensive power is largely harnessed by the official media. Established in 1946, the *People’s Daily* reflects how decision-makers allocated attention to certain topics, and provides a timeline of SO development. Accordingly, in delivering information through comprehensive coverage across approximately 100 columns, this study reflects the will of decision-makers and public opinion and offers a comprehensive, historical, and authoritative perspective from the official media [5]. Therefore, a co-word analysis of the *People’s Daily* reports reveals the evolutionary logic of SOs’ participation in social governance because it offers more than a simple review of policy history or study of focus events. Given that social governance involves the management of social implementation by multiple governance subjects, this analysis acts as a quantitative yardstick of the content and attitudes of the reports over a period of more than 70 years **(Fig 1)**.

## Research design

### Principle of co-word analysis

Co-word analysis is a type of content analysis method that targets a group of (two) words and counts their occurrences in each time period [24]. Subsequently, a cluster analysis is performed on these words, reflecting the distance relationships between them [24], which, in turn, analyzes the change of themes across different stages. As a common method in research, the co-word analysis method is widely accepted and used by various scholars in different academic studies [25, 26].

In this study, the clustering analysis is part of the co-word analyses. SPSS21.0 statistical analysis software was used to obtain and analyze the thematic clustering results. SPSS21.0 can automatically provide the cluster analysis in different stages and distance coefficients of the co-word group. Nevertheless, it is necessary to rename the clusters stage by stage, determine the tendency and subsequently, explain the distance coefficients between word groups, to correspond to the reality.

### Databases and analytical methods

This study used three database platforms—Chinese Academic Resources Discovery Platform of Renmin University of China (1949–2021), the People’s Database (1949–2021), and CNKI’s Newspaper Navigator (2001–2021)—to conduct an advanced search of the term “social organizations” in the *People’s Daily*. The search results were organized into separate documents by year of sources for thematic analysis, including 73 years, 73 documents (1949–2021), and 898 reports.

After collecting the data, this study used co-word and clustering analysis to analyze all titles and content about SO in the *People’s Daily*. The results were used to demonstrate decision-makers’ attention to SOs in China across different time periods by focusing on attention intensity, emotional tendency, and content.

### Analysis

This study mainly used a combination of thematic analysis and content analysis. To begin the analysis, the theme word, which characterizes the core content of a document and helps determine the document category, was developed according to the principle of saturation (i.e., no additional data with which to develop properties of the category were found) [5]. A manual keyword extraction was performed on the title of each report, yielding 73 annual keyword documents for 73 years of reports.

These documents were subsequently used in the word separation analysis, word frequency analysis, and sentiment analysis. These analyses were performed with NVivo 12.0 and ROST word separation (these software were selected because they do not require complex programming and enable the visualization of the study results).

Next, the co-word and clustering analyses were conducted. A far or near relationship matrix (0–1 relationship matrix) of high-frequency theme words was established for each stage; the judgment criterion was whether a pair of high-frequency theme words existed in the same year. Systematic cluster analysis using SPSS 21 was conducted to gather high-frequency words that were closer together and more closely related to each other to form specific word groups. The types of topics that decision-makers emphasized most at certain stages were derived accordingly [27].

Finally, these attention distributions of the clustering maps in each stage were interpreted, and the overall change trend of all stages of clustering maps was derived by the inductive method.

## Results and discussion

### Stage division: Analysis of attention intensity and emotion

**Fig 2:**
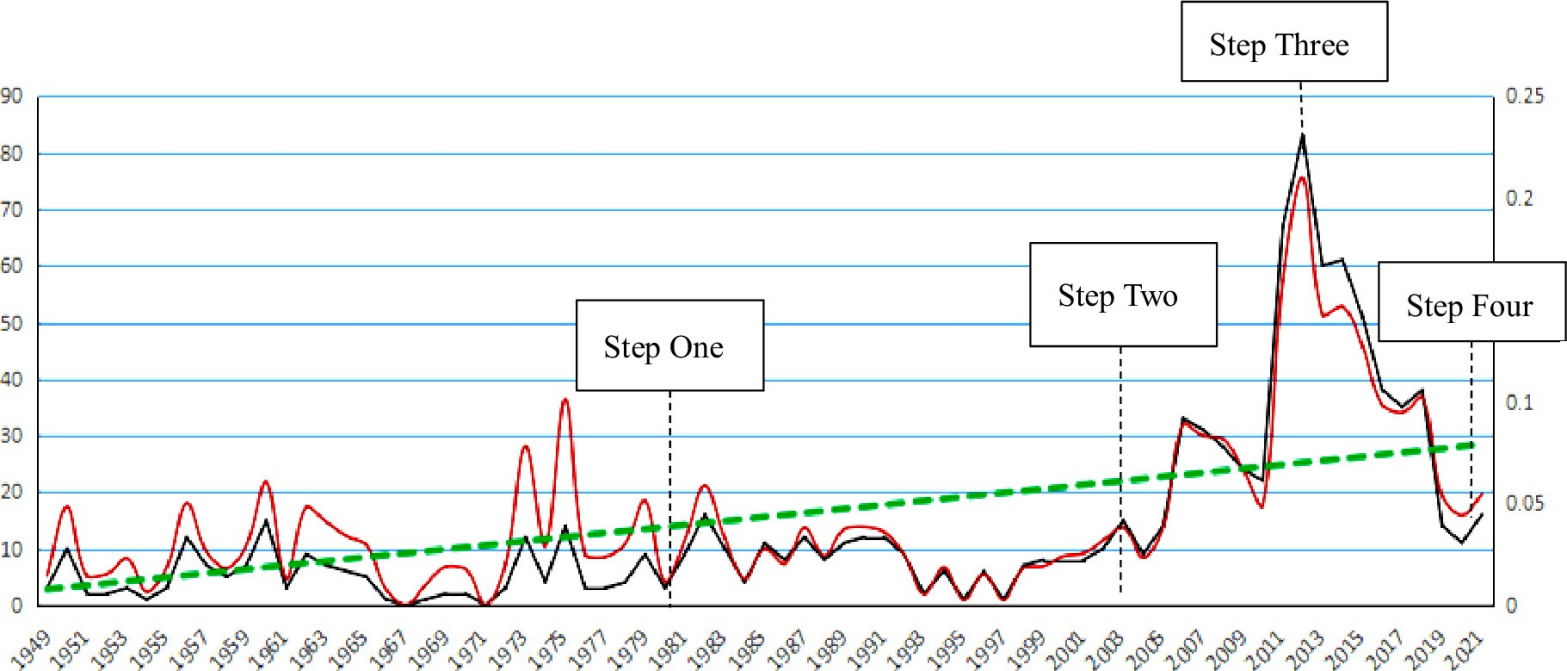
Number and annual percentage of reports on social organizations by People’s Daily. Black line signifies the number of reports on SOs in the *People’s Daily*. Red line denotes the ratio of report number on SOs to all reports. Green line represents the trends of the annual rate of report number.

Corresponding with the results of the number and annual percentage of reports on social organizations by the *People’s Daily* **(Fig 2)**, the overall level of topic distribution intensity for SOs’ participation in social governance in the newspaper was low, representing a mere 0.25% of all news. In terms of emotional tendency, the results revealed a spectrum of single to mixed emotions.

Based on the changes in attention intensity in Fig 2, the distribution of attention to SOs’ participation in social governance from 1949 to 2021 can be divided into four distinct fluctuations based on time periods: large and frequent fluctuations; small and moderate fluctuations; sharp rising fluctuations; and sharp falling fluctuations. Such divisions show that attention intensity was unstable, fluctuated greatly and there was no consensus on the importance of SOs. Notably, attention was scattered in the early stage and concentrated in the sharp falling fluctuations stage. Similarly, using word frequency quantity analysis as well as focus event analysis, this study divided SO history into the following distinct stages.

- During the first stage (1949–1981), the *People’s Daily* attention to SOs was fragmented and unstable. Authoritative media reports could show that SO development was affected by the institutional environment and focus events.
- During the second stage (1982–2003), attention remained relatively limited but was more stable. The Constitution of 1982 had specific provisions on SOs, and the *People’s Daily* reprinted scholars’ discussions on the participation of SOs in public economic life. Thus, 1982 was chosen as a new starting point.
- During the third stage (2004–2011), attention intensity fluctuated sharply and peaked in 2011. Notably, Guo Meimei, a 20-year-old girl who claimed to be “living in a luxury house, driving a Maserati” and had a micro-blog authenticating identity of “Business General Manager of the Red Cross Society of China” attracted national attention, which smeared the Red Cross Society of China and brought China’s public welfare community into disrepute over this scandal.
- During the fourth stage (2012–2021), attention decreased drastically every year, with signs of intermittent recovery in 2014, 2018, and 2021.

**Fig 3:**
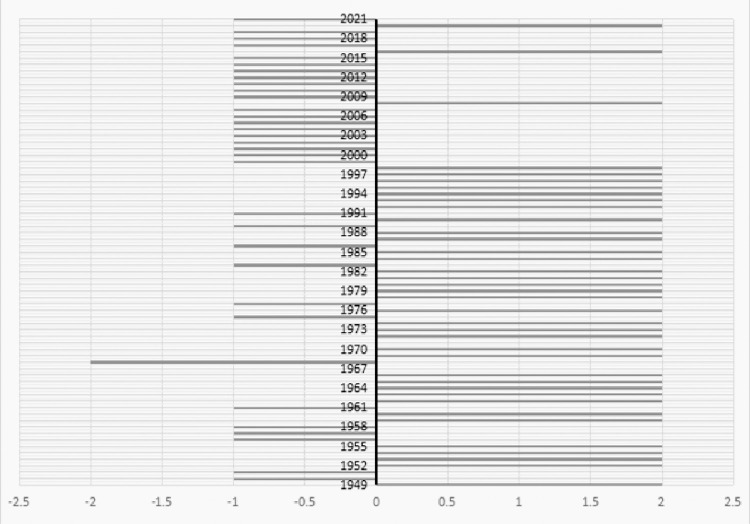
Emotional tendency reported by social organizations in the People’s Daily. Positive emotion assignment value of +2. Negative affective assignment of -2. Mixed emotion assignment of -1.

In terms of attention sentiment variation in **Fig 3**, the trend ranged from extremely positive to negative and mixed coverage. This outcome may be a side-effect of the increasing complexity of matters related to SOs.

- The positive reports were concentrated between 1992 and 1998, when the Jilin Provincial Charity Federation was established, and the Interim Regulations on the Registration and Administration of Private-Run Non-Profit Enterprises were issued.
- The second stage of positive reports was concentrated during 1962–1966, 1978–1982, and 1952–1955. The years 2008, 2016, and 2020 had more positive reports. Extremely negative reports were concentrated in 1968, when the social and economic life reform plan proposed by vice-president Liu Shaoqi was opposed, and in 1967, when Liu was persecuted for political reasons during the Great Proletarian Cultural Revolution. At the time, the participation of SOs in the social governance program that Liu supported was prohibited; subsequently, there were no relevant reports on the SO topic in 1967.
- Mixed emotions were concentrated during 1999–2007, 2009–2015, 2017–2019, and 2021.

### Description of attention distribution: Analysis of attention content and stage changes

#### First stage of attention distribution: The formative period (1949–1981)

**Fig 4:**
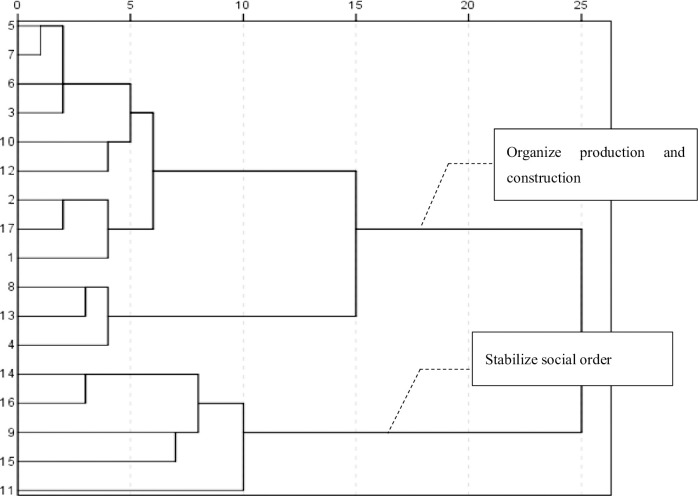
Cluster analysis atlas of high-frequency words from 1949 to 1981.

In the first stage (**Table 1 and Fig 4**), the state attached importance to the two functions of SOs—organizing production (core topic: agricultural mutual assistance and cooperation) and stabilizing social order (core topic: women’s participation). The first word group shows that the SOs had production capacity. Socialist production was required to mobilize SOs—including health organizations (similar to the World Health Organization), agricultural mutual assistance and cooperation groups (farmers help farmers groups), intellectual youth organizations (organizations working to eliminate illiteracy), and transport cooperatives (organizations and groups contributing to road construction)—as the new force helping construct the new China. The word “organize” in the first stage was semantically a verb, such as organizing labor and sports competitions; the coefficients of each high-frequency word during the clustering process were very high, except for “mutual assistance and cooperation in agriculture” and “mobilizing social forces,” indicating that in this stage, there was little consensus on attention to SO, and the focus was scattered.

**Table 1:** Statistics of high-frequency words developed in Chinese social organizations from 1949 to 1981.

	Frequency	Keywords	Frequency	Keywords	Frequency
1. Socialist education	33	Social forces mobilization	8	13. Health organization and revolution	6
2. International community	14	8. Militiaman	7	14. Women’s participation	4
3. Organized production	14	College students’ social survey	7	15. Social reform	4
4. Sports competition	12	10. Intellectual youth organization	7	16. Social order	4
5. Agricultural mutual assistance and cooperation	11	11. Trade union organization	6	17. Mass movement	4
6. Socialist construction	10	Social transportation	6		

The second word group signifies that the stability of socialist transformation was highly valued by the government. Women’s organizations and trade unions were formed to help maintain social order. The government found it convenient for women to conduct their ideological work and improve women’s status, and likewise found that protecting the rights and interests of workers in the process of social reforms, via labor organization and health reforms, was necessary. From 1949 to 1981, SOs comprised one category, covering trade unions and basketball teams. During this time, SOs were active across a variety of fields, primarily in basketball games and railroad transport. However, the concept of SOs was vague and included, for example, public security and sports organizations, militia organizations, railroad cooperatives, and cultural mass organizations in **Table 1**. SOs became a new force for uniting people, carrying out socialist development, and defending the achievements of socialism in the new China. In addition, the state gradually started to focus on SOs and viewed them as “fully utilizing and organizing.” Overall, this stage was the formative period of attention distribution.

#### Second stage of attention distribution: The normative period (1982–2003)

**Fig 5:**
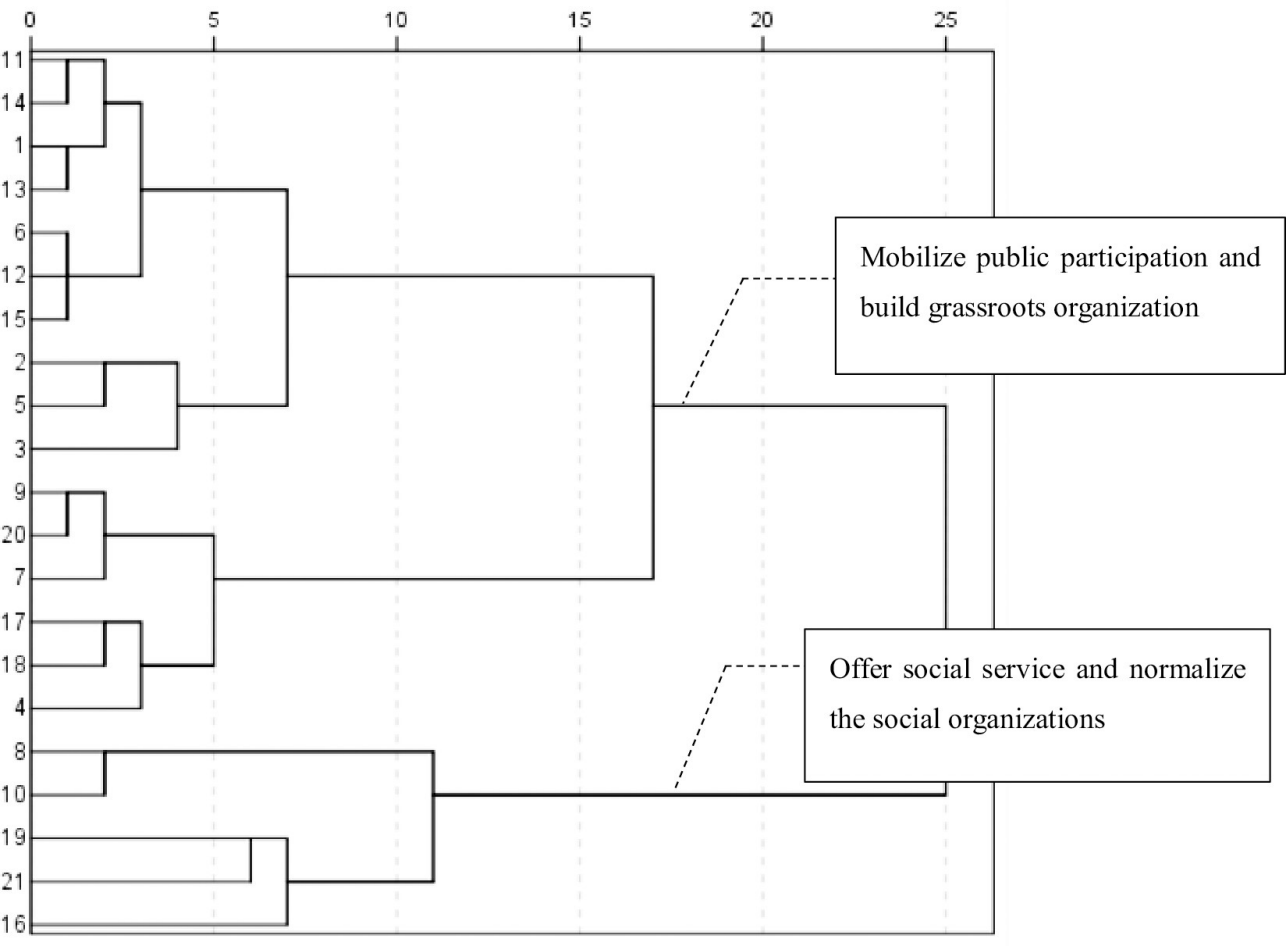
Cluster analysis atlas of high-frequency words from 1982–2003.

In the second stage (**Table 2 and Fig 5**), two SO functions—mobilization and service—received considerable attention, and the SO service was standardized. The first type of theme word group indicated that SOs played an indispensable role in social and economic life, responding to contemporary needs and contributing to the modernization of socialism with Chinese characteristics. During this period, SOs were viewed as tools for helping the government mobilize and organize social resources. The similarity coefficients of high-frequency words in the cluster analysis indicated that SOs’ participation was most closely tied to the security of society, grassroots organization-building, and global SO.

**Table 2:** Statistics of high-frequency words in Chinese social organizations from 1982–2003.

Keywords	Frequency	Keywords	Frequency	Keywords	Frequency
1. International community	23	8. Double support work for veterans	8	15. Social participation mobilization	5
2. Socialism with Chinese characteristics	16	9. Scientific and technological research	8	16. Reform	5
3. Rural autonomy	15	10. Social services	7	17. Social security	4
4. Construction of modernization	12	11. Grassroots organization building	6	18. Organizing production	4
5. Social survey and practice	11	12. Security of society	6	19. Outlawing illegal organizations	4
6. Social and economic life	10	13. Communist Youth League Organization	6	20. Civil organization management	3
7. Underworld organization	9	Global social organization	6	21. Social audit organization	3

During this stage, Chinese official media fully demonstrated attention distribution from an institutional development perspective. First, as a social force, mass self-governing organizations, such as the Grassroots Security Committees, Women’s Federation, and Child Welfare Organization, participated in the security of society and the protection of women’s and children’s rights and interests. These organizations protected grassroots society and special groups. Second, industry associations and social intermediary organizations—such as scientific and technological research organizations—and other civil organizations contributed greatly to grassroots organization-building. Third, international SOs were involved in the maintenance of international relations and negotiations; for example, Beijing’s SOs cooperated with the World Health Organization in 2003 in the fight against pneumonia in Fig 5.

The second type of theme word groups demonstrates that SO reform and social services had become significant topics for the country. Underground organizations were being outlawed (reports on cult organizations during 2001–2002 attracted wide attention and discussion); meanwhile, social services provided by SOs were encouraged, such as dispute mediation and caring for veterans and martyrs’ families by mobilizing businesses and volunteers. Between 1982 and 2003, Chinese SOs were developing and transforming. Nevertheless, attention to SOs was still low and scattered as the concept of SO was not widely understood. This limited, scattered attention existed despite SOs’ active participation in the modernization of rural self-government, scientific and technological research, as well as international and domestic communications. This stage was the normative period of attention distribution.

#### Third stage of attention distribution: The exploratory period (2004–2011)

**Fig 6:**
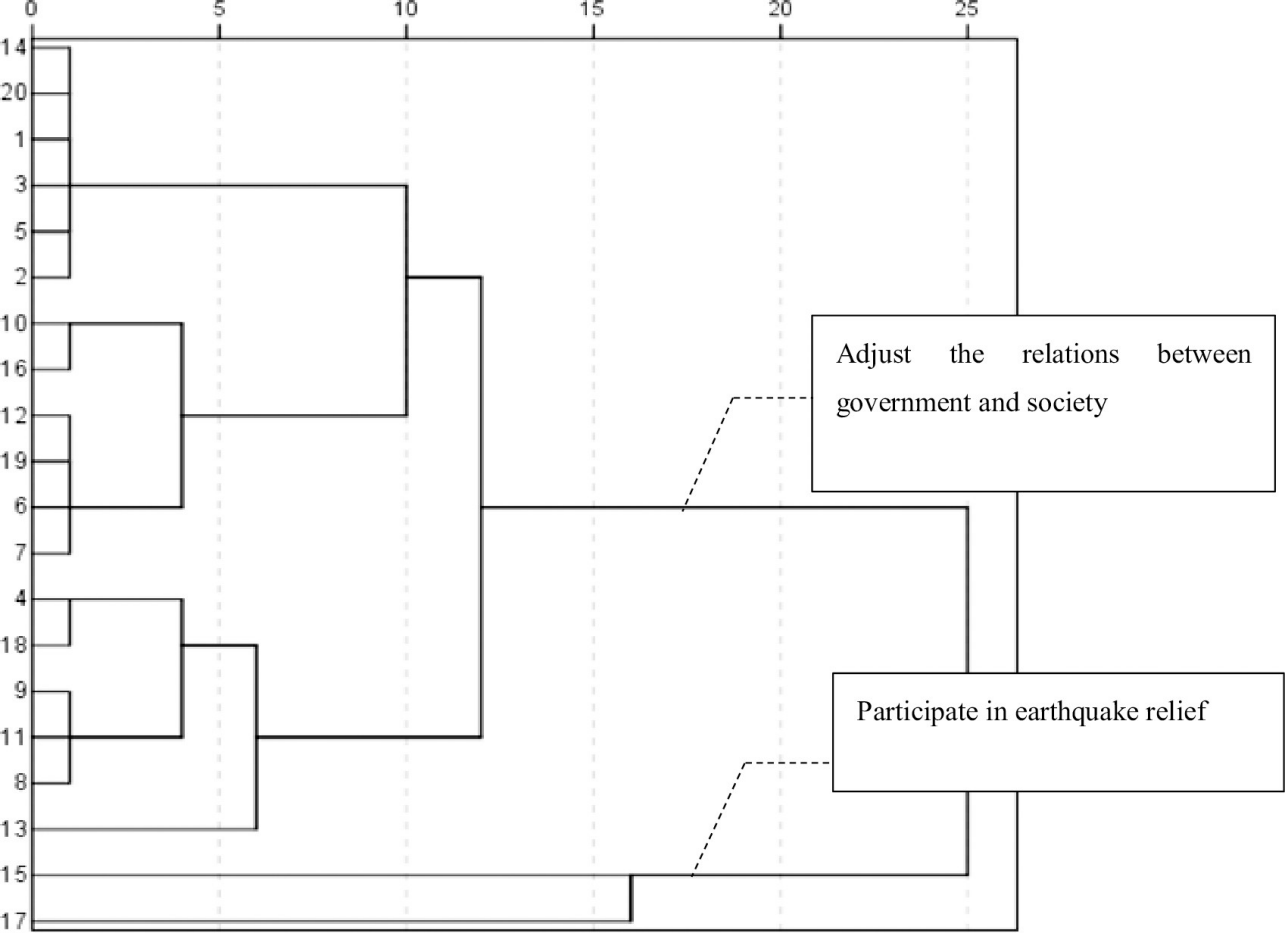
Cluster analysis atlas of high-frequency words from 2004–2011.

During the third stage, the focus was on two topics: “new SOs” and the adjustment of the relationship between government and society. The effect of SOs participating in earthquake relief efforts was also addressed. First, in 2005, the concept of “two new organizations” (new economic organizations and new SOs) was initially reported exclusively by the *People’s Daily*. Its topics showed that new SOs were closely related to the development of a harmonious society in **Table 3**, which is a long-held principle in China. It refers to a stable and orderly society characterized by democracy, a legal system, fairness and justice, honesty and fraternity, creativity, and people and nature living in harmony. Here, the development of a harmonious society is related to the development of the CPC, grassroots organizations, and communities. As China’s socialist market economy developed with its reform and opening-up, various non-governmental SOs, including intermediary organizations, social groups, foundations, private non-enterprise units, and various mass groups emerged. These groups contrasted with more traditional organizations, such as political parties and governments; accordingly, they were referred to as “new SOs.” With the emergence of new rural development organizations and flat communication network organizations, topics related to how SOs participate in social management innovation and adjustments to make government–SO relationships more equal garnered considerable attention. Particularly, in 2004, reports focused on the improvement of public service functions, innovations in classified guidance social management mechanisms, the development of market-oriented intermediary organizations, and coordinating role of industry associations in **Table 3 and Fig 6**.

**Table 3:** Statistics of high-frequency words in Chinese social organizations from 2004–2011.

Keywords	Frequency	Keywords	Frequency	Keywords	Frequency
1. Harmonious society construction	93	8. New rural construction	7	15. Combat the earthquake and carry out relief work	4
2. Study and practice activities	22	9. Social organization function	6	16. Transfer of government functions	4
3. Building of the Communist Party of China (CPC)	18	10. Community construction	6	Non-public sectors of the economy (NPSOE)	4
4. Innovative social management	13	11. Network organization and organizational network	6	18. Government and social relationships	3
5. Economic and social development	11	12. Rectify illegal behavior	6	19. Industry associations	3
6. Grassroots organization building	9	13. International organizations	5	20. New social organization	3
7. Social organization service	8	14. Trade union organization	4		

The second word group revealed that some SOs were committed to mobilizing social forces for earthquake relief. In major disasters such as the Wenchuan City earthquake in 2008 and the Yushu City earthquake in 2010, SOs—especially charitable organizations—impressed society with their mobilization and volunteer actions. From 2004–2011, SOs attracted more positive attention. After 2008, policymakers began to focus on SOs and developed new understandings of their work; accordingly, studies of new SOs and their activities were added on the agendas of research institutions and government agencies, and the relationship between the state and society was re-examined.

#### Fourth stage of attention distribution: The innovation period (2012–2021)

**Fig 7:**
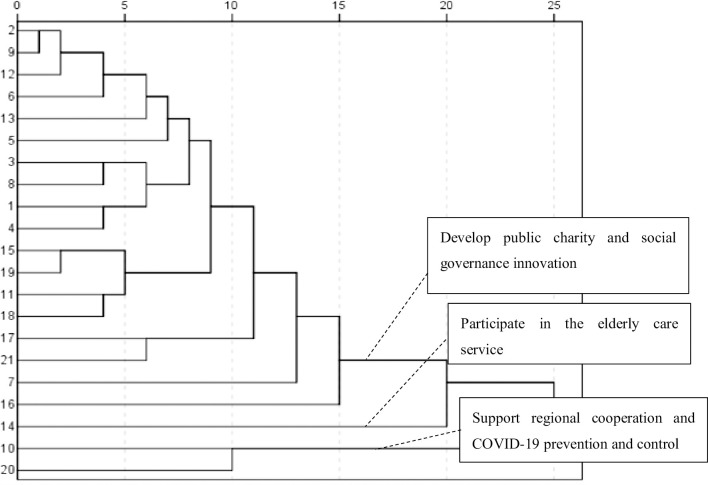
Cluster analysis atlas of high-frequency words from 2012–2021.

In the fourth stage, as shown in **Table 4 and Fig 7**, the attention distribution of official media was more precise, and the topics kept up with the changes occurring that time, implying more diverse attention. The first topic group showed that the field of public welfare charity had become a new focus. The direct registration of SOs started in 2012. The four types of SOs—industry associations and chambers of commerce, science and technology organizations, public welfare and charity organizations, and urban and rural community services—were legally permitted to directly register with the civil affairs department, implying that the dual management system of the registration department and the competent unit had evolved. The first national charity exhibition was held in Shenzhen in July 2012, marking a new era for charitable causes in China. In 2014, an article titled “To Give Charity Its Name Again,” was released, implying that charitable causes were garnering more attention from academia, society, and even the CPC [28]. With the publication of the Charity Law in 2016, in which the scope of charitable organizations was formally described in law for the first time, the concept of “charitable organizations” appeared more, while the concept of “social organizations” appeared less. With this change, the focus shifted to charitable organizations’ participation in social governance innovation and to constraints on charitable organizations. Streamlined administration and institutional decentralization provided more space for the development of SOs, creating the institutional foundation necessary to strengthen participation and innovation in social governance. Meanwhile, the government was the biggest actor in the non-governmental sector—governments at all levels supported healthy and orderly SO development. For instance, the Ministry of Civil Affairs and Yunnan Provincial Government held a “Symposium on Promoting Social Development and Innovation of Social Organizations” in 2013. In 2016, “Opinions on Reforming the Management System of Social Organizations and Promoting Their Healthy and Orderly Development” were issued by the General Office of the CPC, stipulating that, by 2020, a sound management system with a comprehensive registration department and a government department support system (in which each department had its own duties regarding coordination, cooperation, graded responsibility, and supervision according to law) should be established. This crucial task has since been completed.

**Table 4:** Statistics of high-frequency words in Chinese social organizations from 2012 to 2021.

Keywords	Frequency	Keywords	Frequency	Keywords	Frequency
1. Government’s purchase public service (GPPS)	46	8. Community construction	18	15. Grassroots level first	8
2. Healthy and orderly development	34	9. Donation	16	16. Social coordination	6
3. Social governance innovation	30	10. Regional cooperation	13	17. Study	6
4. Building of the Communist Party of China (CPC)	27	11. Registration and cancellation	13	18. Folk	6
5. Charitable cause	25	12. Streamline administration and institute decentralization	10	19. Supervision and norm	6
6. Deep reform	24	13. Illegal social organizations	10	20. Epidemic prevention and control	6
7. International organization exchange	19	14. Participation in aged care service	8	21. Internet	6

The second- and third-word groups revealed that reports on “social organizations playing a role” were more prominent than before, with reports on the purchase of public services, a type of public–private partnership, focusing on services for the elderly and poverty alleviation in tandem with the new areas of epidemic prevention and control in 2020. Hence, SOs’ varied expertise gave them an edge in regional social governance and cooperation. On the one hand, as social governance subjects, SOs can better define social problems and constitute an independent third sector capable of critiquing and supplementing the government’s social governance work. Indeed, 2012–2021 was a period of innovation for charitable organizations; charity supermarkets were fully opened, and citizens could use them to collect, dispose, and sell used items. In addition, joint fundraising by multiple charitable organizations was renovated, online donation became popular, and the field of government-purchased services expanded and escalated. On the other hand, strict regulations of SOs had not decreased; the crackdown on illegal SOs became significantly stronger and civil servants were strictly prohibited from serving in SOs. As the newspaper mentioned, SOs were in a stage of technology upgrading, organizational learning, and cooperation capacity enhancement. Innovative support and vigorous regulations were needed in the joint fight against the COVID-19 pandemic.

### Predicting social organizations’ participation in social governance: An analysis of three evolving characteristics

There are two main factors influencing the attention distribution regarding SOs’ participation in social governance from the perspective of official media and the state: socially-oriented factors (e.g., modernization and technological progress, reform and opening-up, the development of socialist democratic politics [29], and the outbreak of COVID-19) and organizationally-oriented factors (e.g., SO functions and capacity-building). Beyond these factors three major changes were key to SO development.

First, since the founding of the People’s Republic of China (PRC), coverage of SOs has been social and organizational in nature. According to the frequency and topic groups, “social-oriented character” and “organized-oriented character” were evident during SO changes, except in the first stage (1949–1981). In the subsequent stages (1982–2021), the social nature of SOs was reported more frequently than the organizational nature. In terms of social orientation, rural and community organizations came into focus, whereby rural self-governance practices increased to help rural areas create and maintain their services. In terms of organizational orientation, the focus shifted from the development of organizational production to the standardization of SOs, the role of SOs, and innovations related to their mechanisms. In other words, the modernization of SOs and the development of public services and public welfare charities reflected the dual development trend of “meeting social needs” and “regulating organizational structure.”

Second, SO management from the national perspective strengthened the guiding power of the CPC. The relationship between government and society, with respect to political control, shifted. In the first and second stages of attention distribution, SO management was more related to the development of the Communist Youth League, which has, over the past 30 years, assisted more than 3.38 million students from rural areas and built 15,444 primary schools. The third and fourth stages highlighted the importance of party organization-building in the management of SOs, closely relating SOs with grassroots organization-building, CPC building, and learning the CPC’s spirit as well as accepting the CPC’s thought guidance. These findings imply that SOs established themselves as a social force. Their operations absorb and transform the rules of state development, resulting in a benign interaction between the leading political potential of the state and the social momentum of social subjectivity. Accordingly, SOs should clarify the scope of their activities in the context of the CPC’s leadership.

Third, the nature of SOs’ participation in social governance services from the national perspective signifies the drift of organizational goals (i.e., the trend from social mobilization to autonomous charity innovation reveals a narrowing). This change is most obvious in the management and development of SOs, which has gradually shifted focus to public charity. However, this transition demonstrates a certain degree of target drift. Semantically, the percentage of “social organizations” in the overall coverage decreases annually, while alternative expressions, such as “multi-governance, grassroots governance, actor networks, community service organizations, and charitable organizations,” increase annually. The coverage focuses on public service purchase, from management to governance, CPC-building in SOs, public and private charities, and collaborative community innovation. Additionally, non-profit organizations participated in social governance mechanism innovation. Overall, there seems to be a disproportionate focus on the structure, governance, and mechanisms of these organizations compared to their activity, philosophy, and effect. Political factors (e.g., the CPC leads everything) and social-cultural factors (e.g., collectivist culture) in China are reflected in the topic term clustering results for the official media. Notably, focused SO functions subject to central oversight contribute to national stability. While there is a certain goal drift for SOs (i.e., emphasis on offering public services and neglecting the organization of collective action), there is also the possibility that SOs will gradually be replaced by other kinds of organizations, such as charitable organizations or foundations.

## Conclusions, explanations, and policy suggestions

### Conclusions

Traditional Chinese society has historically homogenized the family and state, meaning people interact with and comprise only two kinds of organizations: the family and governmental institutions. Consequently, the concept of SO did not exist prior to the reform. However, after the reform and opening-up, SOs gradually became one of the multiple subjects in social governance [30]. Academic discussions on the evolution of SOs and the state–society relationship focus on two concerns: whether society can supervise the government and how society can facilitate the state’s social governance [31]. Considering SOs’ development from the state perspective complements “social initiative theory” by reflecting on two aspects: (1) the social nature of SOs and the adjustment of the relationship between government and society; and (2) the degree of organization and regulation of SOs. Over time, attention distribution changed from social mobilization in the first two phases (“Formative-Normative Period”) to philanthropic innovation in the second two phases (“Exploratory-Innovative Period”).

**Fig 8:**
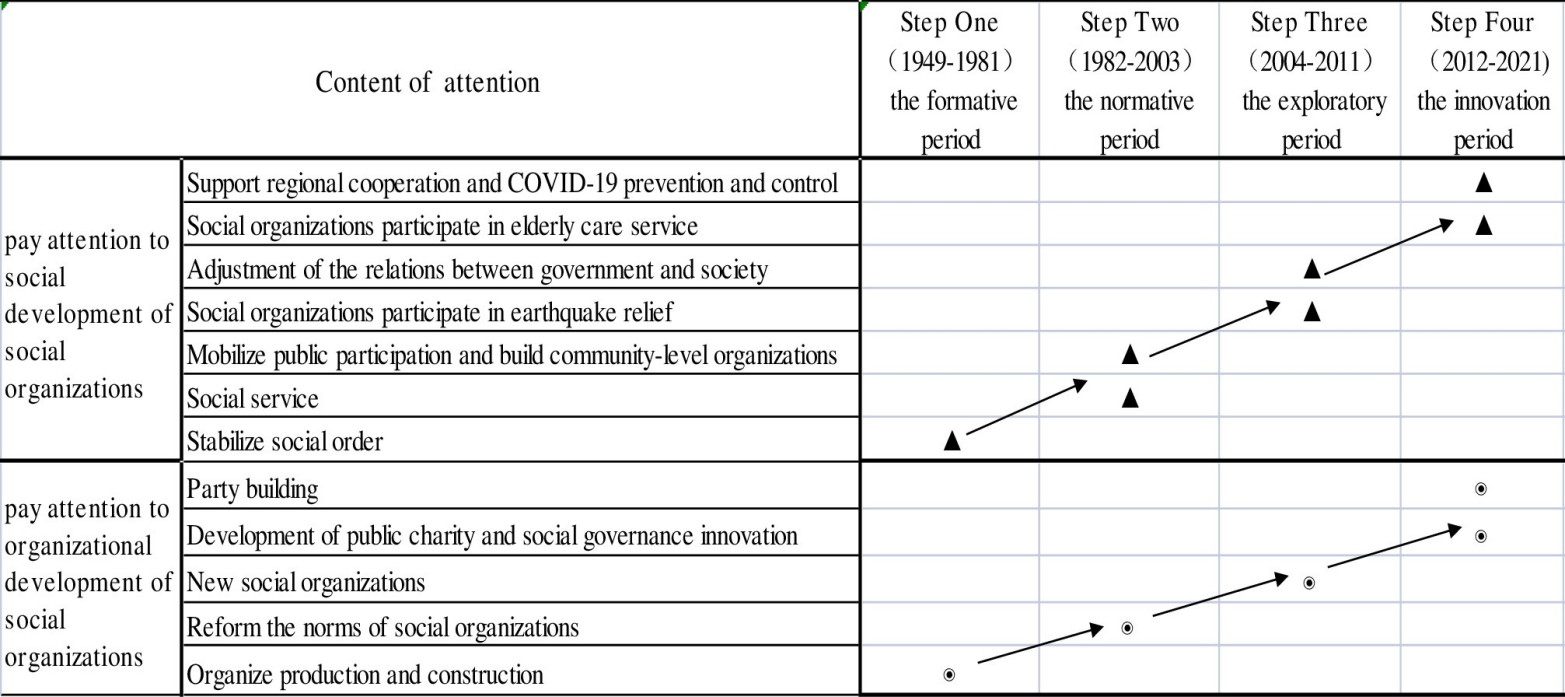
Changing attention distribution of social organizations participating in social governance.

**Fig 9:**
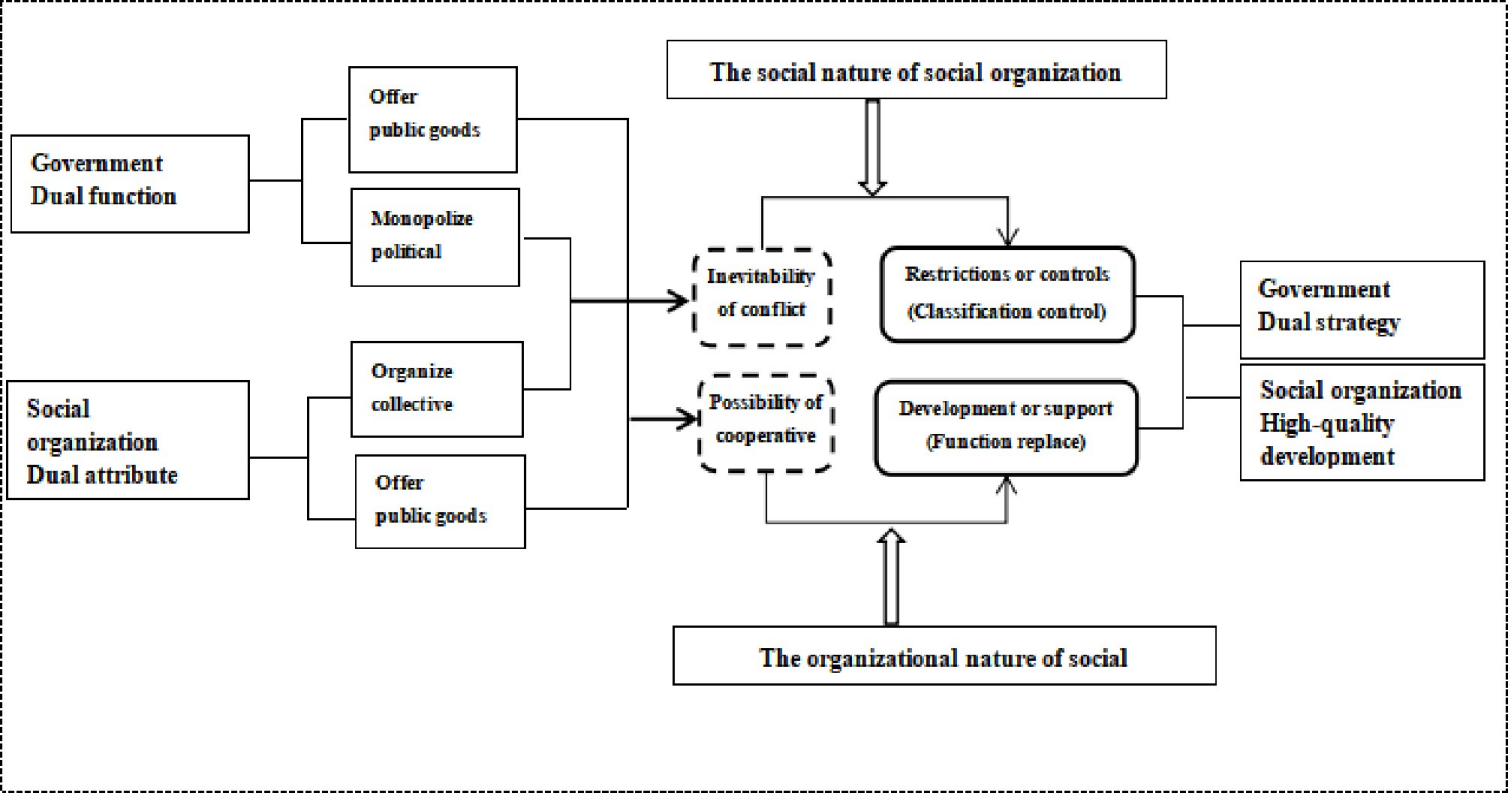
Theoretical contribution of using institution development to observe the history of social organizations in the official media.

In contrast to existing studies, this study partially supports the classification divided by “policy instruments” [10] as **Table 5**, and fully supports the two attributes of “the inevitability of contradiction in organizing collective action” and “the possibility of cooperation in offering public goods” proposed by the AAS theory. Specifically, this theory states that governments conducted the dual strategy of classification control and function replacement, which shows a real relationship between government and society as **Fig 8** [32]. Accordingly, studies on SO management and development should use the institution development perspective to better observe SOs’ history in the official media as **Fig 9**. The two lines of social-orientation and organizational-orientation—empirically support and supplement the AAS theory. The relationship between government and society is characterized by the government’s dual function (offering public goods and monopolizing political power) and SOs’ dual attributes (organizing collective action and offering public goods) [32].

**Table 5:** Focus event research results.

Name	Standard of Focus Event	Stage Division	Stage Content
Wang [14]	State policy of reform and opening up	Dichotomic	1978, before/ after reform and opening-up
Fang [13]	Social structure under the imperial system—political party transformation—macro management standardization	Trisection	Before 1978, before the founding of People’s Republic of China (PRC) 1949–1978, after the founding of PRC 1979–present, the reform and opening-up
Li & Liang [12]	Strategic direction adjustment—response adjustment—interaction between the government and social organization	Quartered	1949–1965, beginning of the founding of PRC 1966–1977, during the Cultural Revolution 1978–2000, after the reform and opening- up 2001–present, since the 21st century
Wang [17]	Technology development dimension	Quartered	Before 1949, traditional social organizations in China 1949–1977, current social organizations in China 1978–1999, social organizations in the new era 2000–present, network social organizations
Zhou & Li [11]	Focus event of the development of significant social organizations	Quartered	1949–1980, period of historical stagnation 1981–1993, period of contemporary recovery 1994–2007 period of rapid development 2008–2016 period of contemporary transition
Li, Cheng, and Jia [18]	Relations between government and SO: “classified control,” “regulatory control,” and “empowerment control”	Quartered	1950–1988, “identity trust” stage 1989–2011, “clean up and rectify” stage 2012–2017, “combination of discharge and management” stage
Wang [10]	Management and control strength dimension; promotion of development dimension	Quartered	1949–1977, only authoritative policy tools 1978–2003, development-like tools start to emerge 2004–2011, the proportion of development tools used increases 2012–present, promoting social governance is emphasized in the “development” dimension
Luo [36]	Cooperative relationship between the country, the market, and the society	Quartered	1949–1956, unity-type social organization 1956–1978, administrative social organization 1978–2012, exploration stage of governance-oriented social organizations 2012–present, a formed governance social organization
This paper (2023)	Institutional construction Perspective and attention distribution	Quartered	1949–1981, the formative period 1982–2003, the normative period 2004–2011, the exploratory period 2012–present, the innovation period

Furthermore, the other two new findings also support and supplement the AAS theory. There was the gradual strengthening of the main leading power, which shifted from the Communist Youth League of China to the CPC, which shows a stronger absorbing capacity of the government, implying that the government can support SOs to act in the areas it specifies; for example, the government will buy more services from SOs. Additionally, SO goals shifted from conducting general functions to innovation related to special charitable organizations, which verified that the government conducts a dual strategy of classification control (i.e., restrictions or controls on SOs posing a threat to the state order) and function replacement (i.e., developing or supporting SOs that can help the government provide public goods). Meanwhile, SOs demonstrated a high-quality development strategy of providing support in the areas permitted by the state as well as improving and innovating themselves (Fig 9). Therefore, SO development has gradually concentrated on public services and public charity, emphasizing “public welfare” [33–35].

### Explanation of the changes in political and social relations in the four stages of attention distribution

The attention distribution of state and official media to the management and development of SOs has undergone four stages of change: formative, normative, exploration, and innovation periods. There are profound reasons behind these changes, such as the process factor of participation in social governance, institutional environment factor, and organizational function factor, which produce changes in the government–SO relationship.

Consequently, the SO concept has narrowed from civil society organizations to charities and contracted public services. In the first stage, SOs were viewed as trade unions, women’s organizations, agricultural cooperatives, sports organizations, and so on. The second stage expanded the definition of SO in the context of “socialism’s social public economic life” [37], distinguishing SOs from state institutions and enterprises in terms of their classifications, roles, and funding sources. Although these SOs lacked the functions of economic organization and management, they had a sizable mass base. The third stage introduced the concept of “new SOs” and “new economic organizations” as well as narrowing and clarifying the scope of non-governmental organizations emerging from this period. Consequently, these “new SOs” were often related to intermediary organizations, social groups, foundations, and private non-enterprise units. In the fourth stage, SOs were recognized as one of the main subjects of social governance; the government still played a positive interventionist role. During this stage, registration regulations of the Ministry of Civil Affairs and 2016 Charity Law set SO boundaries, limiting them to foundations, social groups, and social service organizations.

Beyond the government factor, the social environment influenced the development of SOs, or, more specifically, socioeconomic and political activities, through social governance institutions and structures. This, in turn, affected how SOs participated in social governance. On the one hand, the role of political system factors was the most direct and obvious [38]. The co-word analysis revealed high-frequency word pairs with high correlations, such as grassroots organization development and SO service, public welfare charity and care services for the elderly, regulating illegal behavior and civil society organizations, social governance innovation and public environmental litigation, and social governance innovation and national fitness. The social environment has shaped the institutional development perspective; accordingly, the focus event perspective cannot clarify the above situation. On the other hand, the adjustment of relations between the government and SOs is structural, reflecting modern social-economic life. After the reform and opening-up, decision-makers had a new understanding of the power and role of social organizations. For example, the second stage focused mostly on civil and international SOs.

The last major reason for the changed government–SO relationship derives from the degree to which SO capacity-building affects the state’s attitude toward SOs. The Third Plenary Session of the Twelfth Central Committee of the CPC explicitly requires that public services and other suitable matters be entrusted to SOs. Accordingly, it proposes the autonomy requirement of “three points, five self and four no’s” for SOs. Specifically, there were three points to change: (1) advance the current system reform by implementing a system of government–society and management–office separation and by reversing the original “dual-department” management system; (2) ensure SOs’ independence by adhering to the “five-self” principle (voluntary initiative, self-selected president, self-raised funds, self-employed staff, and self-help meetings) and the “four no’s” principle (no administrative level, no official establishment, no competent business department, and no current national staff working part-time); and (3) increase government support for the development of SOs and experiment with new modes of government–society collaboration. The development of SOs is largely dependent on their own functions and capacity-building, which is the internal strength that determines the relationship between the government and SOs.

### Contributions and the prospect of policy formulation

There are four insights into policy formulation regarding SOs that correspond to the analysis of the mechanism of “participation in social governance process—institutional environment—organizational function.” First, there is a need to increase policy-making initiatives, mobilize social forces as the main positive actor for participation in social governance, and encourage autonomous philanthropic innovation. Second, policy-making needs to implement digital governance and define SO. Third, policy-making related to SOs needs to respect their organizationally-oriented and socially-oriented natures of development. Fourth, to produce new knowledge, policy-making must emphasize the role of official media and encourage conducting big data and data mining analyses on continuous thematic stories for multiple years.

This study is novel in two aspects. The first is regarding its method; specifically, this study applied a new method, co-word analysis, to narrate a long-time Chinese story of SO participation in social governance from a novel view, the institutional development perspective. This perspective offers a more accurate measure of the reality of SO participation in social governance because it involves a mixed research methodology using continuous data from 73 years of reports and content mining, as well as topic clustering analysis to reveal a macroscopic and multi-line picture. Notably, topic clustering analysis and closely paired topics can provide insights for future causal mechanism analysis. This study’s analysis accumulated evidence on “China’s Way of Governance” and offered a basis for modern social governance policy innovation.

The second novel aspect relates to the content of the analysis. Topic distribution in official media replaced the focus event perspective and supplemented the AST, historically used by researchers on this topic, and the AAS theory. In China, the narrative represented by the official media is very typical and prominent. By extension, other countries can examine the development history of SOs participating in social governance in the national narrative to predict the national development trend of SOs. Therefore, a constructive perspective needs to be added to the natural order of the general media to consider official media-setting—in addition to public agenda-setting, media agenda-setting, and policy agenda-setting—in the AST. Additionally, this study verified the AAS theory using two lines of topic clustering trends (Fig 8). Accordingly, the research of “attention distribution in official media from an institutional development perspective” is relevant in today’s scenario and has substantial implications for the field and society at large. This study has optimized the role of official media reports in analyzing the national attention distribution and in applying novel big data mining analysis methods to determine the social and organizational nature of SOs.
